# “*Candidatus* Intestinibacterium parameciiphilum”—member of the “*Candidatus* Paracaedibacteraceae” family (*Alphaproteobacteria*, *Holosporales*) inhabiting the ciliated protist *Paramecium*

**DOI:** 10.1007/s10123-023-00414-5

**Published:** 2023-08-24

**Authors:** Olivia Lanzoni, Franziska Szokoli, Martina Schrallhammer, Elena Sabaneyeva, Sascha Krenek, Thomas G. Doak, Franco Verni, Thomas U. Berendonk, Michele Castelli, Giulio Petroni

**Affiliations:** 1 Department of Biology, University of Pisa, Pisa, Italy; 2 Institut für Hydrobiologie, Technische Universität Dresden, Dresden, Germany; 3 Mikrobiologie, Institut für Biologie II, Albert-Ludwigs-Universität Freiburg, Freiburg, Germany; 4 Faculty of Biology, Saint Petersburg State University, Saint Petersburg, Russia; 5 Biology, Indiana University, Bloomington, IN USA; 6 Department of Biology and Biotechnology, University of Pavia, Pavia, Italy

**Keywords:** *Paramecium*, *Paracaedibacteraceae*, Bacterial endosymbionts, Ciliate protists, Symbiosis, *Holosporales*

## Abstract

Protists frequently host diverse bacterial symbionts, in particular those affiliated with the order *Holosporales* (*Alphaproteobacteria*). All characterised members of this bacterial lineage have been retrieved in obligate association with a wide range of eukaryotes, especially multiple protist lineages (e.g. amoebozoans, ciliates, cercozoans, euglenids, and nucleariids), as well as some metazoans (especially arthropods and related ecdysozoans). While the genus *Paramecium* and other ciliates have been deeply investigated for the presence of symbionts, known members of the family “*Candidatus* Paracaedibacteraceae” (*Holosporales*) are currently underrepresented in such hosts. Herein, we report the description of “*Candidatus* Intestinibacterium parameciiphilum” within the family “*Candidatus* Paracaedibacteraceae”, inhabiting the cytoplasm of *Paramecium biaurelia*. This novel bacterium is almost twice as big as its relative “*Candidatus *Intestinibacterium nucleariae” from the opisthokont *Nuclearia* and does not present a surrounding halo. Based on phylogenetic analyses of 16S rRNA gene sequences, we identified six further potential species-level lineages within the genus. Based on the provenance of the respective samples, we investigated the environmental distribution of the representatives of “*Candidatus* Intestinibacterium” species. Obtained results are consistent with an obligate endosymbiotic lifestyle, with protists, in particular freshwater ones, as hosts. Thus, available data suggest that association with freshwater protists could be the ancestral condition for the members of the “*Candidatus* Intestinibacterium” genus.

**Supplementary Information:**

The online version contains supplementary material available at 10.1007/s10123-023-00414-5.

## Introduction

Symbiotic associations between protists and bacteria are rather common in nature, and examples can be found in almost every protist group (Gast et al. 2009; Nowack and Melkonian 2010; Dziallas et al. 2012; Scheid 2014; Husnik et al. 2021; Fokin and Serra 2022). Different kinds of associations were observed, ranging from temporary to rather evolutionary stable. While the breadth of the diversity of such association is still undisclosed, their outcomes may enhance the possibility to adopt novel lifestyles and colonise new environments, otherwise not accessible for the protist (Fenchel and Finlay 1991; Görtz and Fokin 2009; Dziallas et al. 2012).

Many bacterial symbionts of diverse protists are affiliated to the alphaproteobacterial order *Holosporales* (sensu Szokoli et al. 2016a), which was initially considered as early divergent within the order* Rickettsiales*, but, as now indicated by multiple studies, is a fully independent lineage within *Alphaproteobacteria* (Martijn et al. 2018; Muñoz-Gómez et al. 2019; Castelli et al. 2022b). It has also been proposed that *Holosporales* (sensu Szokoli et al. 2016a) should be down-ranked to the family level, namely *Holosporaceae *(*Rhodospirillales*) (Muñoz-Gómez et al. 2019). Here, the definition of *Holosporales* (sensu Szokoli et al. 2016a) will be used, considering that it best highlights the evolutionary distinctiveness of this lineage, as well as the broad diversity of its family-level sublineages.

At present, Holosporales comprise exclusively bacteria associated with different eukaryotes and can be subdivided into four families: *Holosporaceae* (*sensu* Szokoli et al. 2016a), “*Caedimonadaceae*” (Schrallhammer et al. 2018), “*Candidatus* (from now on, abbreviated as *Ca*.) Hepatincolaceae” (Szokoli et al. 2016a), and “*Ca*. Paracaedibacteraceae” (Hess et al. 2016).

Holosporaceae comprise many endosymbionts of ciliate protists, in particular of the genus *Paramecium*. The most renowned are *Holospora* and the *Holospora*-like bacteria (HLB) (Schrallhammer and Potekhin 2020), which typically reside inside the host nuclear apparatus, displaying a multiphase dimorphic infectious cycle (Beliavskaia et al. 2020; Schrallhammer and Potekhin 2020; Zilio et al. 2021). *Holosporaceae* hosts include other protists (e.g. diplonemids), and even arthropods.

“*Caedimonadaceae*” as well include many bacteria associated with protists. Among these, the most deeply investigated is “*Caedimonas varicaedens*” (Schrallhammer et al. 2018; Flemming et al. 2021), which was shown to confer to its *Paramecium* hosts a competitively advantageous killer trait towards non-infected conspecifics (Schrallhammer and Schweikert 2009).

“*Ca*. Hepanticolaceae” (Szokoli et al. 2016a), also termed “*Ca*. Tenuibacteraceae” (Kroer et al. 2016), were detected up to now in association with multicellular animals, specifically with Ecdysozoa (Wang et al. 2004; Kroer et al. 2016; Guidetti et al. 2020).

The family “*Ca*. Paracaedibacteraceae” includes numerous symbionts of unicellular eukaryotes. Earlier studies were mainly focused on *Acanthamoeba* spp. (Amoebozoa), found to be hosting members of the paraphyletic genus “*Ca*. Paracaedibacter” (Horn et al. 1999) and “*Ca*. Odyssella thessalonicensis” (Birtles et al. 2000), as well as on uncharacterised protists from acidic environments, hosting “*Ca*. Captivus acidiprotistae” (Baker et al. 2003). More recent investigations on phylogenetically diverse hosts allowed the description of “*Ca*. Finniella spp.”, inhabiting Viridiraptoridae (Cercozoa) (Hess et al. 2016) and ciliates (Boscaro et al. 2019), of “*Ca*. Intestinibacterium nucleariae” (originally denominated “*Ca*. Intestinusbacter nucleariae”) (Dirren and Posch 2016; Oren et al. 2020), described in the opisthokont *Nuclearia* (Holomycota), and of “*Ca*. Parafinniella ignota”, hosted by the ciliate *Euplotes* (Boscaro et al. 2019). Notably, the kinetoplastid *Bodo saltans* (Euglenozoa) is possibly dependent on its *Paracaedibacter*-like bacterium “*Ca*. Bodocaedibacter vickermanii” (Midha et al. 2021).

For what concerns ciliate protists, despite intensive investigations on bacterial symbionts (e.g. Hirakata et al. 2015; Schuster and Bright 2016; Boscaro et al. 2019; Takeshita et al. 2019; Serra et al. 2020; Graf et al. 2021; Muñoz-Gómez et al. 2021; Castelli et al. 2022a), only few records on “*Ca*. Paracaedibacteraceae” bacteria were recently reported (Boscaro et al. 2019). Thus, according to such data, “*Ca*. Paracaedibacteraceae” seem to be underrepresented in ciliates as compared to other families of *Holosporales*. Herein, we present the description of a novel bacterial symbiont occupying the cytoplasm of *Paramecium biaurelia* (Ciliophora, Oligohymenophorea), and affiliated to “*Ca*. Paracaedibacteraceae”.

## Material and methods

### Host cultivation and characterisation

The *Paramecium* strain US_Bl 12I1 was isolated from a water sample obtained during an environmental survey around Bloomington, Indiana (USA), in 2011. The sample originated from the Skater’s pond (39°14′40″N, 86°32′30″W) located approximately 10 km outside Bloomington. A monoclonal culture of the ciliate was established by single-cell isolation, then maintained at 22 ± 1°C in 0.25% Cerophyl medium inoculated with *Raoultella planticola* strain DMSZ 3069. Medium was prepared according to Krenek et al. (2011), namely by an infusion of wheatgrass powder (GSE Vertrieb GmbH). *Paramecium* species was preliminarily identified by live observations as a member of the *Paramecium aurelia* species complex according to the morphological criteria by Fokin (2010), namely cell size and shape, number, type and structure of contracting vacuoles, and number and location of micronuclei (applying a DAPI stain). Then, identification was confirmed by molecular characterisation using three markers. In detail, total DNA was extracted from approximately 50 *Paramecium* cells using the NucleoSpin® Plant DNA Extraction Kit (Macherey-Nagel GmbH & Co. KG, Düren NRW, Germany) as described elsewhere (Szokoli et al. 2016a). The eukaryotic SSU rRNA gene was amplified and sequenced according to Modeo et al. (2013); the internal transcribed spacer (ITS) region and the mitochondrial cytochrome *c* oxidase subunit 1 (COI) gene were amplified and sequenced as in Lanzoni et al. (2016). PCR products were purified with the EuroGold CyclePure Kit (EuroClone S.p.A. Headquarters & Marketing, Pero, Milan, Italy). Sanger sequencing was performed by GATC Biotech AG (Konstanz, Germany). The sequences of the SSU rRNA gene and ITS were then joined together by the partial overlap on the SSU rRNA gene portion, as described previously (Sabaneyeva et al. 2018).

### Molecular characterisation of the symbiont and fluorescence in situ hybridisation

The prokaryotic SSU rRNA gene sequence of the bacterial symbiont of *Paramecium* US_Bl 12I1 was obtained by a touchdown PCR with the *Alphaproteobacteria*-targeted forward primer 16Sα_F19b and the almost universal bacterial reverse primer 16S_R1522a, as described previously (Szokoli et al. 2016b). PCR products were purified and sequenced using internal primers (16S F343 ND 5′-TACGGGAGGCAGCAG-3′, 16S R515 ND 5′-ACCGCGGCTGCTGGCAC-3′, and 16S F785 ND 5′-GGATTAGATACCCTGGTA-3′) as previously described (Szokoli et al. 2016b).

Based on the obtained sequence, the genus-specific oligonucleotide probe IntGen_189 [5′-GCGGTAAACCTTTAACCTC-3′] (Cy3-labelled) and the species-specific probe IntPar_79 [5′-CTAACATATAGAGCAAGCTCC-3′] (Cy3-labelled) were designed and then synthesised by Eurofins GmbH (Ebersberg, Germany). In silico probe specificity was determined using the TestProbe tool 3.0 of the SILVA rRNA database project (Quast et al. 2013) and the probe match tool of the Ribosomal Database Project (RDP) (Cole et al. 2009), and by manual inspection of sequence hits. The probes were tested in fluorescence in situ hybridisation (FISH) experiments in combination with the almost universal bacterial probe EUB338 (Amann et al. 1990) (FITC-labelled) at different formamide concentrations (from 0 up to 50%) for their binding ability on the US_Bl 12I1 symbiont, following the protocol by Szokoli et al. (2016a). Observations were carried out with a Leica TCS SPE confocal laser scanning microscope (Leica Microsystems GmbH, Wetzlar, Germany) in the Core Facility Center for Microscopy and Microanalysis (St. Petersburg State University).

### Transmission electron microscopy

For transmission electron microscopy, *Paramecium* cells were fixed for 1.5 h at room temperature in a mixture of 1.6% PFA and 2.5% glutaraldehyde in 0.1 M phosphate buffer (pH 7.2–7.4). Then, the cells were washed in the same buffer containing sucrose (12.5%) and post-fixed for 1 h at 4°C in 1.6% OsO_4_. Dehydration of the cells was performed in an ethanol gradient followed by ethanol/acetone (1:1), 100% acetone, and the cells were finally embedded in Epoxy embedding medium (Fluka Chemie AG, St. Gallen, Switzerland) according to the manufacturer’s protocol. The blocks were sectioned with a Leica EM UC6 Ultracut, and sections were stained with aqueous 1% uranyl acetate followed by 1% lead citrate. All samples were examined with a JEM-1400 electron microscope (JEOL, Ltd., Tokyo, Japan) at 90 kV. The images were obtained with a built-in digital camera (Nitla et al. 2019).

### Phylogenetic analyses of the symbiont

Phylogenetic analyses were performed using the ARB software package version 5.2 (Ludwig et al. 2004). The novel sequence of the endosymbiont was automatically aligned together with the nearly full-length (>1200bp) sequences of 59 other Holosporales and of six other *Alphaproteobacteria*, representing the outgroup. The alignment was later manually refined according to the predicted secondary structure of the SSU rRNA and trimmed at both ends to the length of the shortest sequences, obtaining 1451 final positions. The optimal substitution model (GTR+I+G) was determined with jModelTest 2.1 (Darriba et al. 2012) using the Akaike Information Criterion (AIC). Maximum likelihood (ML) phylogenetic analysis was performed with 1000 bootstrap pseudo-replicates with the PhyML software (Guindon and Gascuel 2003) version 2.4.5 from the ARB package. Bayesian inference (BI) was performed with MrBayes 3.2 (Ronquist et al. 2012), using three runs, each with one cold and three heated chains, iterating for 1,000,000 generations with a burn-in of 25%. Convergence was determined by reaching an average deviation of split frequency below 0.01, and potential scale reduction factor (PSRF) for all parameters close to 1.000. Pairwise identity values between sequences were calculated on the same matrix employed for phylogeny.

### Screening of metagenomic datasets

The online platform IMNGS (Integrated Microbial Next Generation Sequencing) (Lagkouvardos et al. 2016) was used to perform a systematic screening of the sequences from all available prokaryotic SSU rRNA gene amplicon studies available in the Sequence Read Archive (SRA). The sequences of the herein characterised bacterial symbiont from *Paramecium* strain US_Bl 12I1 and its close relative “*Ca*. Intestinibacterium nucleariae” inhabiting *Nuclearia delicatula* (Dirren and Posch 2016) (accession number: LN875069) were used as queries, applying a 95% similarity threshold, as described by Lanzoni et al. (2019).

Afterwards, as described by Lanzoni et al. (2019), in order to investigate the environmental distribution, the obtained IMNGS hits were classified as environmental sequences (i.e. freshwater, seawater, anthropogenic, soil) or as derived from potentially host-associated bacteria (i.e. crustaceans, fish, nematodes, plants, poriferans, unicellular). Two indices were employed to assess the environmental distribution of bacteria related to the herein described US_Bl 12I1 symbiont and those related to its close relative “*Ca*. Intestinibacterium nucleariae”, namely “frequency of occurrence” and “relative abundance” for each environmental category, following Lanzoni et al. (2019). Briefly, frequency of occurrence was calculated as the number of samples which resulted positive for the presence of the bacterium versus the total investigated samples assigned to the environmental category, whereas the relative abundance was obtained by calculating for each sample the ratio between the number of sequences assigned to the bacterium and the total number of sequences present in the same sample, and then averaging this values for the samples of the same environmental category.

## Results

### Molecular identification of host and symbiont

The US_Bl 12I1 strain was identified as *Paramecium biaurelia*, having 99.9% or higher sequence identity with published *P. biaurelia* sequences on NCBI in the joined partial SSU rRNA gene - ITS - partial LSU rRNA gene sequence (2798 bp, accession number: KX712111), and 99.0% identity in the COI gene sequence (760 bp, accession number: KX712112), while identity value in the latter gene dropped down to 79–86% with respect to other species of the *P. aurelia* complex.

The SSU rRNA gene sequence of the symbiont (1284 bp, accession number: KX702973) was closely related to “*Ca*. Intestinibacterium nucleariae” (sequence identity of 97.8%; accession number: LN875069; Dirren and Posch 2016) and to a number of environmental sequences obtained from metagenomic studies (e.g. DQ336985: 97.7%, HE797838: 94.9%) in a BLASTn search on NCBI Nucleotide collection. Thereafter, taking into consideration that SSU rRNA gene identity with “*Ca*. Intestinibacterium nucleariae” is below the threshold established to distinguish different bacterial species (98.65–98.7%) (Stackebrandt and Ebers 2006; Kim et al. 2014b), and above the genus threshold (94.5%) (Yarza et al. 2014), we considered the herein characterised symbiont as a representative of a novel species of the genus “*Ca*. Intestinibacterium” (Dirren and Posch 2016). It will be referred to as “*Ca*. Intestinibacterium parameciiphilum” from now on (see taxonomic description at the end of the “Discussion” section).

### Symbiont 16S rRNA gene phylogeny

**Fig. 1:**
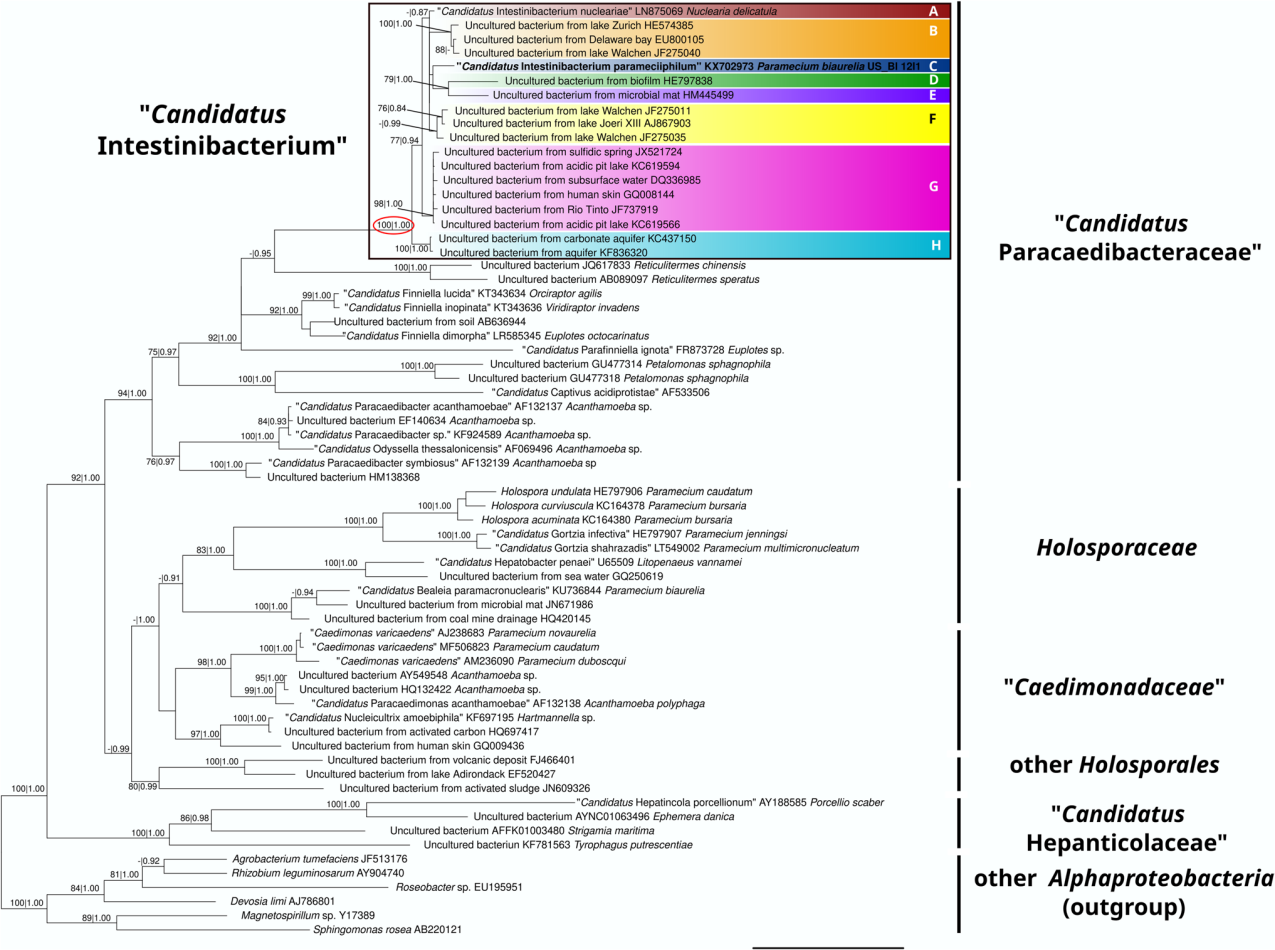
Bayesian inference tree based on 66 prokaryotic SSU rRNA gene sequences, namely 60 *Holosporales* and six other *Alphaproteobacteria* as outgroup, showing the phylogenetic position of the *Paramecium* symbiont US_Bl 12I1 labelled as “*Ca*. Intestinibacterium parameciiphilum” (in bold). Major lineages, including families of *Holosporales*, are shown on the right side. The “*Ca*. Intestinibacterium” genus clade is enclosed in a black square box, with its support values encircled in red, while its eight subclades are evidenced by colours. Numbers on branches indicate maximum likelihood bootstrap values with 1,000 pseudoreplicates and Bayesian posterior probabilities after 1,000,000 iterations (values below 70|0.80 were omitted). The scale bar stands for an estimated sequence divergence of 10%

The endosymbiont of *Paramecium* strain US_Bl 12I1 is clustered within the “*Ca*. Paracaedibacteraceae” family. Specifically, it formed a fully supported (100 ML|1.00 BI) clade together with “*Ca*. Intestinibacterium nucleariae” and several other sequences of different environmental origins (“*Ca*. Intestinibacterium” clade, enclosed in a black square box in Fig. 1). Within this clade, all sequence identities were above the commonly accepted genus threshold for the 16S rRNA gene of 94.5%, while they were much lower with other representatives of “*Ca*. Paracaedibacteraceae” (Supplementary Table 1). Thus, the representatives of this lineage were assigned to the “*Ca*. Intestinibacterium” genus (Fig. 1).

The phylogenetic relationships within “*Ca*. Intestinibacterium” were not always reconstructed with high support. Nevertheless, based on sequence identities, eight species-level (sub)clades (A–H) could be identified. While the relationships among clades were not fully resolved, all clades encompassing more than a single organism were quite highly supported (all ≥0.99 BI, all except one ≥98 ML; Fig. 1). Specifically, sequence identities within each clade (≥99.4%; Supplementary Table 1) were always well above the established species-level threshold of 98.65–98.7% (Stackebrandt and Ebers 2006; Kim et al. 2014b), while identities between sequences from different clades were below the threshold, with only very few exceptions (none of which pertaining the newly characterised symbiont of *P. biaurelia*).

Clade A is constituted by “*Ca*. Intestinibacterium nucleariae”, symbiont of *Nuclearia delicatula* (Dirren and Posch 2016). Clade B includes uncultured bacteria isolated from freshwater lakes, plus a sequence from the marine Delaware Bay (EU800105) (Shaw et al. 2008). Clade C is formed by the novel “*Ca*. Intestinibacterium parameciiphilum” symbiont of *P. biaurelia*. Each of clade D and clade E is constituted by a single sequence, respectively, a bacterium from biofilm (HE797838) (Kim et al. 2014a) and another one isolated from microbial mat of lava tube walls (HM445499) (Marshall Hathaway et al. 2014). Clade F contains bacterial sequences derived from freshwater lakes (Yuhana et al. 2006; Jogler et al. 2011) and drinking water (Gomez-Alvarez et al. 2015). Clade G consists of uncultured bacteria retrieved from different sources, such as a terrestrial sulphidic spring (JX521724) (Headd and Engel 2014), subsurface water of the Kalahari Shield (DQ336985) (Gihring et al. 2006), an acidic pit lake from the Iberian Pyrite Belt (KC619566) (Santofimia et al. 2013), the river Rio Tinto (JF737919) (García-Moyano et al. 2012), and even a human skin sample (GQ008144) (Grice et al. 2009; García-Moyano et al. 2012). Finally, clade H consists of bacteria derived from aquifers (KC437150; KF836320) (Moser, D.P., et al.; Reihle J. et al.; unpublished data).

### Environmental distribution

**Fig. 2:**
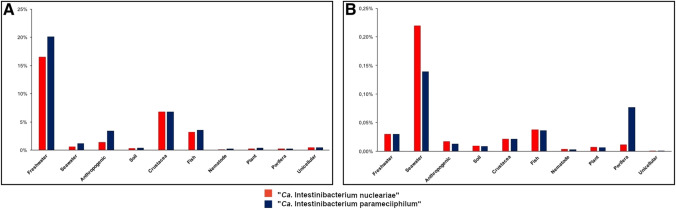
Environmental distribution of the *Paramecium* symbiont US_Bl 12I1 “*Ca*. Intestinibacterium parameciiphilum” and its close relative “*Ca*. Intestinibacterium nucleariae”. (**A**) Frequency of occurrence calculated as the number of positive samples on the total number of samples available for the respective environmental category; (**B**) relative abundance expressed as the average ratio between positive hits and the total number of sequences within each sample of the category. On the x axis, the environmental categories and potential host organisms are represented, whereas in the y axis the frequency of occurrence and relative abundance are shown, respectively

The environmental screening showed that the distribution and abundance of sequences related to “*Ca*. Intestinibacterium parameciiphilum” and those related to “*Ca*. Intestinibacterium nucleariae” are partly comparable in terms of the two indices employed (see methods for details) (Fig. 2). Specifically, the highest frequency of occurrence (indicative of the environmental diffusion of symbionts’ relatives) was found in freshwater environments, exceeding 20% and 15%, respectively (Fig. 2A). In other environments, the hits related to each of the two symbionts showed a lower frequency of occurrence, down to below 3% for seawater, soil, and anthropogenic environments (Fig. 2A). Despite the high frequency of occurrence in freshwater samples, the relative abundances (indicative of the richness of symbionts’ relatives) in positive samples were very low (Fig. 2B). The same trend of very low frequency of occurrence was observed in all the other environments (Fig. 2), with the relatively highest abundances reached in seawater (0.22% and 0.14%, for “*Ca*. Intestinibacterium nucleariae” and “*Ca*. Intestinibacterium parameciiphilum”, respectively; Fig. 2B).

Concerning potential associations with eukaryotes, most common were those with crustacean and fish samples (for both queries, frequency of occurrence over 5% and 3% respectively; Fig. 2A). In general, relative abundances were low in all samples associated with potential hosts (Fig. 2B), with a comparatively slightly higher abundance in crustaceans and fishes than, for instance, in nematodes and plants. However, the highest relative abundance (nearly 0.08%) was found in association with porifers for relatives of “*Ca*. Intestinibacterium parameciiphilum”, while the one of “*Ca*. Intestinibacterium nucleariae”–related sequences in the same hosts was proportionally much lower (0.01%; Fig. 2B).

Interestingly, a sample of human skin microbiome (SRP056364) (van Rensburg et al. 2015) showed more than 1% of relative abundance for “Ca. Intestinibacterium parameciiphilum” (data not shown).

### Probe design and fluorescence in situ hybridisation experiments

The specificity of the newly designed genus- and species-specific probes, namely IntGen_189 and IntPar_79, was preliminary verified in silico. With 0 mismatches, IntGen_189 hit a number of sequences (50 in RDP, 14 in SILVA), which, based on manual inspection, all resulted to be members of the genus “Ca. Intestinibacterium”, thus confirming the full specificity of the probe (0 filtered non-specific hits; Table 1). Inspected cases include a transcript sequence derived from the chrysophyte *Dinobryon* (Beisser et al. 2017), and representing a putative chimaera between a “*Ca*. Intestinibacterium” bacterium and a *Rhizobium*-like one associated with the chrysophyte.

**Table 1:** In silico matching of probes against prokaryotic SSU rRNA gene sequences available from RDP (release 11, update 5) and SILVA (release 138.1) databases. The reported numbers indicate the non-specific target sequences (i.e., after manual inspection and identification of those affiliated to “*Ca*. Intestinibacterium”) matching the probe with 0 or 1 mismatches

Probe name	Probe sequence	RDP		SILVA	
		0 mismatches	1 mismatch	0 mismatches	1 mismatch
IntGen_189	5′-GCGGTAAACCTTTAACCTC-3′	0	0	0	0
IntPar_79	5′-CTAACATATAGAGCAAGCTCC-3′	0	10	0	6

**Fig. 3:**
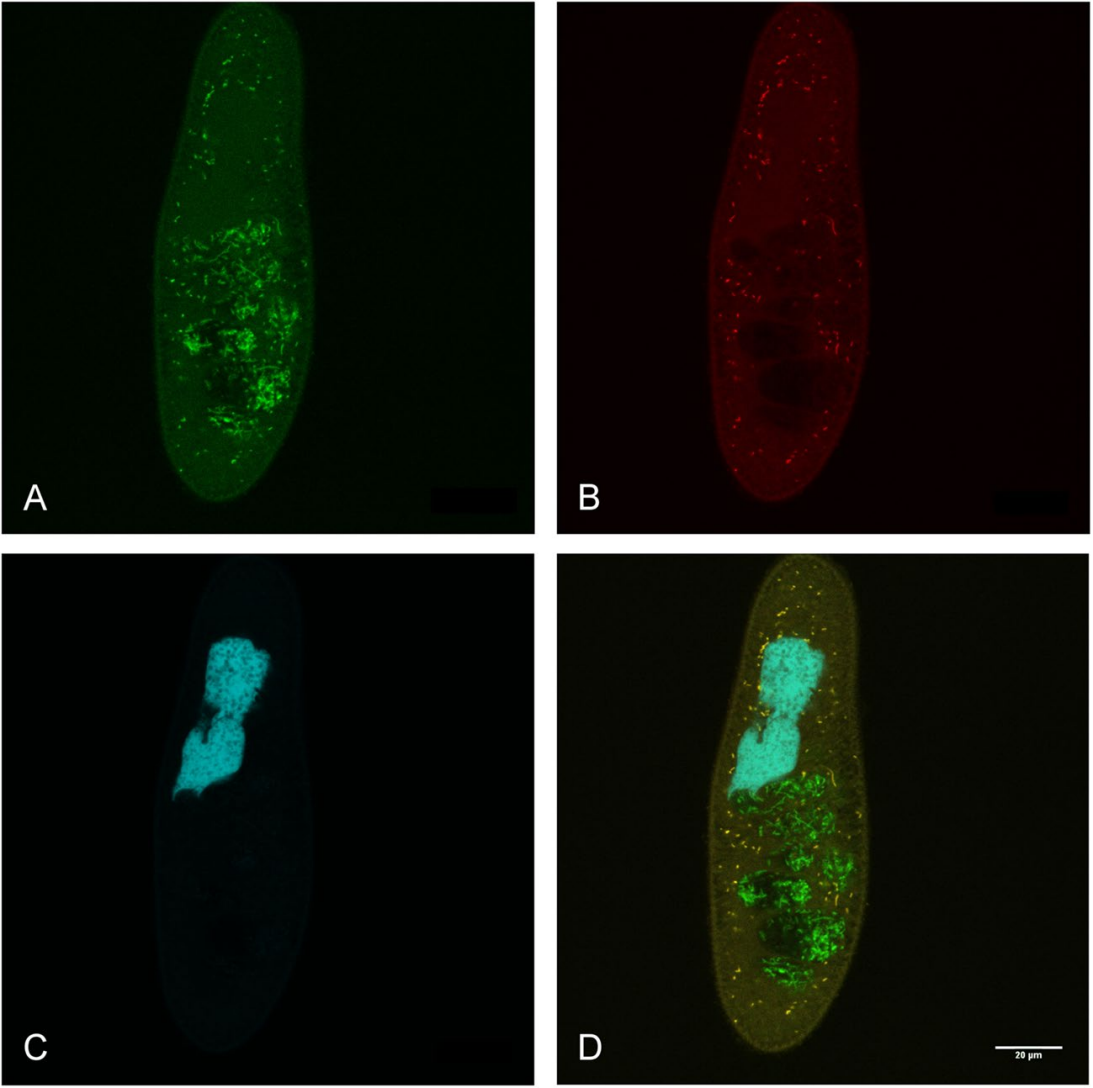
Fluorescence in situ hybridisation of the bacterial symbiont of *Paramecium biaurelia* strain US_Bl 12I1. *Bacteria-*probe EUB338 (FITC-labelled, green signal) targeting bacteria present in the cytoplasm and in food vacuoles of the host cell (**A**); genus-specific probe IntGen_189 (Cy3-labelled, red signal) targeting the symbiont (**B**); DAPI signal (**C**); merge of the three signals (**D**). In the host cytoplasm, symbionts appear yellowish, whereas food bacteria in vacuoles are labelled in green. Scale bar: 20 μm

Allowing 1 mismatch, three additional target sequences were identified on both RDP and SILVA, which were phylogenetically affiliated to “*Ca*. Intestinibacterium” as well, thus confirming a high probe specificity. In experimental tests on the symbiont of *P. biaurelia* US_Bl 12I1, the optimal formamide range for this genus-specific probe IntGen_189 was between 0 and 10% (Fig. 3).

The species specificity of the probe IntPar_79 for the novel symbiont was confirmed as well, as it did not match any other published sequence both in RDP and SILVA in case of 0 mismatches (Supplementary Table 1), whereas, when 1 mismatch was allowed, it matched just four closely related sequences from the same genus (in both RDP and SILVA), plus some *Firmicutes* (i.e. six in RDP and two in SILVA). In the experimental tests, this species-specific probe worked well in 15% formamide.

**Fig. 4:**
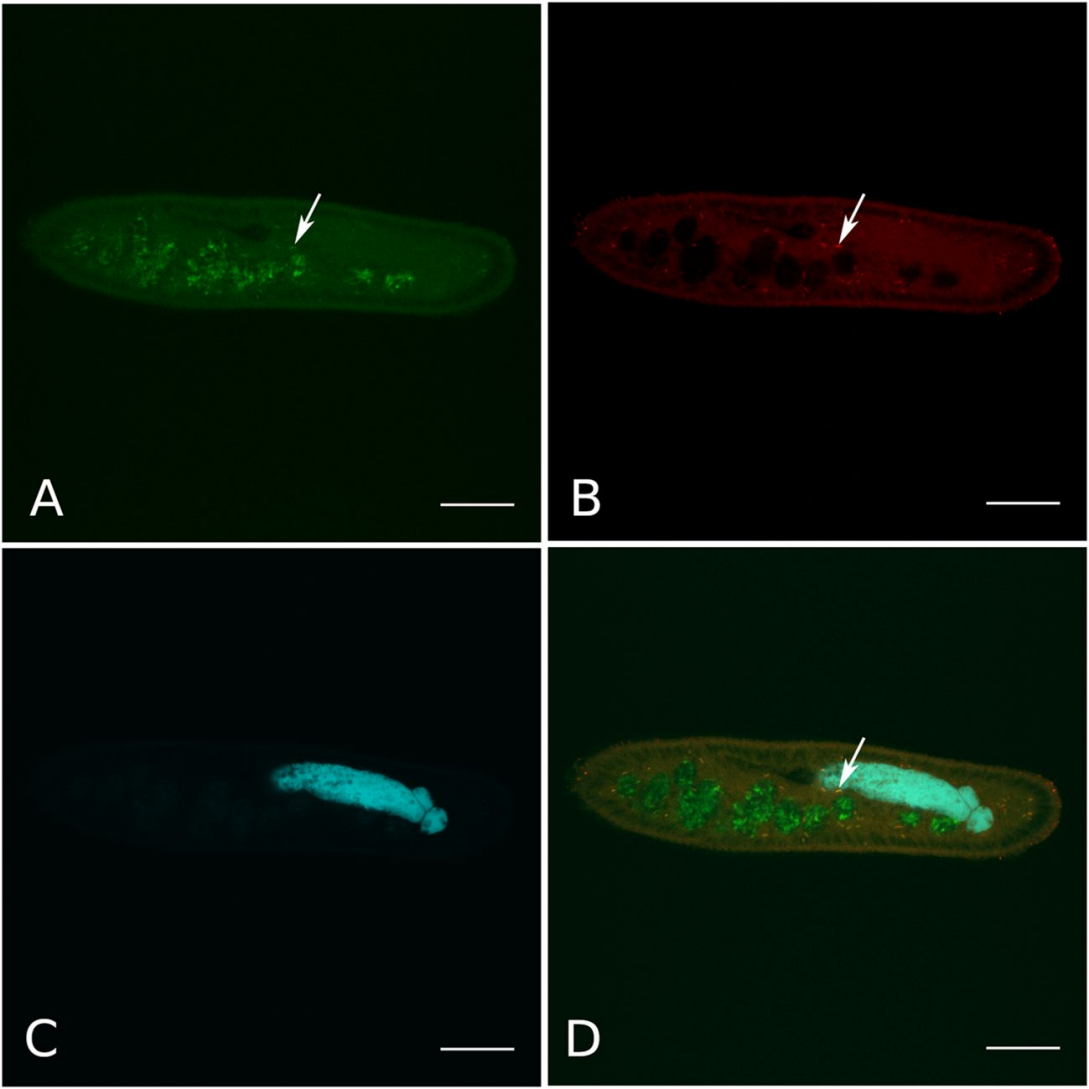
Fluorescence in situ hybridisation of the bacterial symbiont of *Paramecium biaurelia* strain US_Bl 12I1. *Bacteria-*probe EUB338 (FITC-labelled, green signal) targeting bacteria present in the cytoplasm and in food vacuoles of the host cell (**A**); species-specific probe IntPar_79 (Cy3-labelled, red signal) targeting the symbiont (**B**); DAPI signal (**C**); merge of the three signals (**D**). In the host cytoplasm, symbionts appear yellowish, whereas food bacteria in vacuoles are labelled in green. Scale bar: 20 μm

FISH experiments employing the newly designed probes confirmed the presence of the endosymbiont within the cytoplasm of *P. biaurelia* US_Bl 12I1 (Figs. 3 and 4). Bacterial symbionts were never detected in vacuoles with food bacteria (Fig. 3D). Additionally, the signal of each symbiont-specific probe and the almost universal bacterial probe EUB338 fully corresponded in the host cytoplasm, indicating the absence of symbionts other than “*Ca*. Intestinibacterium” (Figs. 3 and 4).

### Endosymbiont ultrastructure

**Fig. 5:**
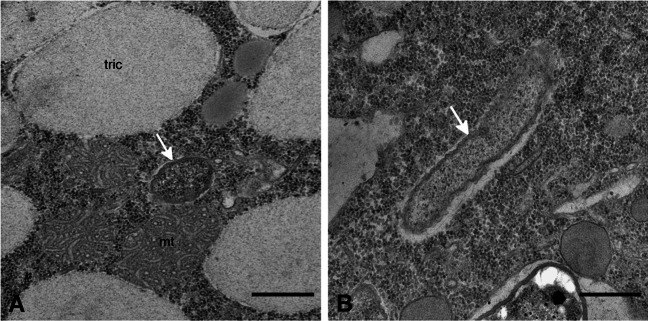
Transmission electron micrographs of *Paramecium biaurelia* isolate US_Bl 12I1. The endosymbiont “*Ca*. Intestinibacterium parameciiphilum” US_Bl 12I1 (white arrow) in transverse (**A**) and longitudinal (**B**) section; “tric” stands for trichocyst; “mt” stands for mitochondrion. Scale bars: 0.5 μm

A single morphotype of intracellular bacteria was observed in the cytoplasm of *P. biaurelia* US_Bl 12I1. These symbionts showed the typical membrane organisation of Gram-negative bacteria. They reached 1.50–2.00 μm in length and 0.35–0.38 μm in diameter (Fig. 5) and were evenly distributed inside the host cytoplasm (Figs. 3 and 5). Neither flagella nor a host-derived membrane surrounding the bacteria was observed. We also did not register any inclusions or virus-like particles in the bacterial cytoplasm (Fig. 5). In contrast to “*Ca*. Intestinibacterium nucleariae” (Dirren and Posch 2016), the *Paramecium* symbiont was not found to be surrounded by a distinct electron-lucent halo (Fig. 5).

## Discussion

In this work, we characterised the bacterial endosymbiont of the *P. biaurelia* strain US_Bl 12I1, which represents one of the few known members of “*Ca*. Paracaedibacteraceae” inhabiting a ciliate host, the others being, to our knowledge, “*Ca*. Finniella dimorpha” and “*Ca*. Parafinniella ignota”, hosted by *Euplotes* spp. (Boscaro et al. 2019). All the other previously characterised representatives of this family (Hess et al. 2016) are endosymbionts of various other lineages of protists, including amoebozoans, cercozoans, and euglenids (Horn et al. 1999; Birtles et al. 2000; Baker et al. 2003; Kim et al. 2010; Dirren and Posch 2016; Hess et al. 2016). The closest described relative of the newly characterised bacterium is “*Ca*. Intestinibacterium nucleariae”, endosymbiont of the nucleariid amoeba *N. delicatula* (Dirren and Posch 2016). The 16S rRNA gene identity with “*Ca.* Intestinibacterium nucleariae” (and with other environmentally derived sequences included in the analysis) is above the threshold established to distinguish different bacterial genera (94.5%) (Yarza et al. 2014) and below the species threshold (98.65–98.7%) (Stackebrandt and Ebers 2006; Kim et al. 2014b) (Supplementary Table 1). Accordingly, the US_Bl 12I1 symbiont belongs to a novel species of the same genus, which we named “Ca. Intestinibacterium parameciiphilum” (see taxonomic description at the end of the “Discussion” section).

“*Ca*. Intestinibacterium parameciiphilum” colonises the cytoplasm of its *Paramecium* host (Figs. 3 and 4), similarly to “*Ca*. Intestinibacterium nucleariae” in *Nuclearia* (Dirren and Posch 2016). Nevertheless, the novel endosymbiont species is twice as big as its relative (1.50–2.00 × 0.35–0.38 μm in size) and does not present a defined white halo around the cell (Fig. 5). Differences in cell morphology have been already described among closely related species of bacterial endosymbionts belonging to the order *Holosporales* (Schrallhammer and Potekhin 2020). Indeed, *Holospora* spp. display variations in cell dimensions and in ultrastructure, possibly connected with the host species and with differences in the bacterial life cycle (Lanzoni et al. 2016). Among symbiotic bacteria in ciliates, another comparable case of variations among congeneric organisms was described for the representatives of the genus “*Ca*. Megaira” (*Rickettsiales*), which showed differences in cell size as well as some morphological peculiarities (Lanzoni et al. 2019). In the case of “*Ca*. Intestinibacterium”, no information is currently available on the causes of such morphological variability. Non-mutually exclusive hypotheses could imply a purely genetic (interspecific) basis, environmental influences (e.g. host-derived effects), or the presence of yet unidentified different stages in the endosymbionts’ life cycle. Further genomic and experimental studies may help in clarifying such features.

Consistently with sequence identities, “*Ca*. Intestinibacterium parameciiphilum” US_Bl 12I1 formed an independent clade with respect to the “*Ca*. Intestinibacterium nucleariae” in the full-length 16S rRNA gene phylogeny (Fig. 1). Moreover, the analysis revealed a much wider phylogenetic diversity within the fully supported (100 ML|1.00 BI) “*Ca*. Intestinibacterium” genus (Fig. 1). Accordingly, we tentatively identified eight sublineages within this genus (clades A–H), each with quite high support (BI≥0.99; ML mostly ≥98), and with identity values overall consistent with the commonly accepted identity thresholds (Stackebrandt; Kim et al. 2014b). Thus, besides the previously described species “*Ca*. Intestinibacterium nucleariae” (clade A) and the novel “*Ca*. Intestinibacterium parameciiphilum” (clade C), each of the other clades could represent a further species, which may be validated by future in-depth studies taking advantage of additional markers, in particular from genome sequences. Those additional “*Ca*. Intestinibacterium” clades include sequences of uncharacterised bacteria derived from various sources, mostly from freshwater, in particular lakes (e.g. Jogler et al. 2011; Santofimia et al. 2013), as well as rivers (García-Moyano et al. 2012), aquifers, subsurface water (Gihring et al. 2006), and springs (Headd and Engel 2014) (Fig. 1). Additional sources include marine environments (Shaw et al. 2008), microbial mats (Marshall Hathaway et al. 2014), and biofilm (Kim et al. 2014a).

According to the screening of the IMNGS database (Fig. 2), the representatives of the genus “*Ca*. Intestinibacterium” are widespread in freshwater environments, in particular, sequence hits for “*Ca*. Intestinibacterium parameciiphilum” were retrieved in over 20% of the investigated freshwater samples (Fig. 2A). A high frequency of occurrence (both for “*Ca*. Intestinibacterium nucleariae” and “*Ca*. Intestinibacterium parameciiphilum”) was found in samples derived from crustaceans and fish (~7% and ~3% respectively; Fig. 2A), and, at least for “*Ca*. Intestinibacterium parameciiphilum”, also from anthropogenic samples (~3% respectively; Fig. 2A). On the other hand, the frequency of occurrence in the other environmental samples or potential hosts was always below 3%, and did not show any evident trend (Fig. 2A).

On average, the relative abundances of “*Ca*. Intestinibacterium” in investigated samples were all very low (<0.25%) in all environments and potential hosts, and in most cases rather comparable between the two species used as queries. The highest levels were observed in seawater samples (more than 0.2% and 0.1% for “*Ca*. Intestinibacterium nucleariae” and “*Ca*. Intestinibacterium parameciiphilum” hits, respectively; Fig. 2B), while the respective frequencies of occurrence in the same environment were rather low (at most ~1%; Fig. 2A).

In terms of range and variability of the two environmental distribution indices, the obtained results are rather consistent with the expectations for symbionts that are obligatorily associated with eukaryotic hosts, and quite comparable to similar investigations on other bacteria with such lifestyles (Lanzoni et al. 2019). In particular, the quite low relative abundances in different environments can be seen as consistent with associations with microscopic hosts (e.g. unicellular eukaryotes), which might have been “trapped” in small amounts during the preparation of samples for environmental 16S rRNA gene metabarcoding studies. This is consistent with the only two characterised cases within the genus “*Ca*. Intestinibacterium”, which are indeed associated with freshwater protists, namely *N. delicatula* and *P. biaurelia* (Dirren and Posch 2016; present work). In parallel, quite high frequencies of occurrence might be explained by the wide environmental distribution of such micro-eukaryotic hosts (Foissner and Hawksworth 2009; Oliverio et al. 2020; Burki et al. 2021).

For what concerns potential associations with metazoans, the quite low relative abundances might find multiple explanations. On one hand, they might be due to the presence of unnoticed microscopic eukaryotes, for example, parasitic ones, which were shown to potentially harbour typically intracellular bacteria such as *Rickettsiales* (Zaila et al. 2017). Alternatively, the bacteria could be actually intracellularly associated with the metazoan cells, as known for other *Holosporales* (Nunan et al. 2013), but with an overall low abundance, which would be consistent with the expectations on the complexity of microbial communities associated with animals (McFall-Ngai et al. 2013; Nogueira et al. 2022; Boscaro et al. 2022).

Interestingly, although only few 16S amplicon sequence hits were overall found in association with humans (Fig. 2), a single sample from human skin (SRP056364) resulted to be quite rich in “*Ca*. Intestinibacterium” (>1% relative abundance) (van Rensburg et al. 2015), and one 16S rRNA gene full-sequence record affiliated to the genus is also derived from human skin (GQ008144) (Grice et al. 2009). Even in these cases, the retrieval of “*Ca*. Intestinibacterium”–related sequence hits might be due to an at least incidental unrecorded occurrence of commensal/parasitic protists (Morán et al. 2013; Magaña et al. 2008; Prieto-Granada et al. 2010), hosting these bacteria as symbionts. Further investigations will be necessary to clarify this point. In any case, considering that the original studies were focused on the composition and role of skin microbiome in healthy and diseased subjects (Grice et al. 2009; van Rensburg et al. 2015), it would be interesting to identify the relative contribution of such potentially unnoticed protists and their putative symbiotic bacteria.

Besides “*Ca*. Intestinibacterium”, other uncharacterised members of the family “*Ca*. Paracaedibacteraceae” may be tentatively assigned to putative hosts based on their provenance, e.g. *Hydra* sp. (Fraune and Bosch 2007), *Apostichopus japonicus* (Zhao et al. 2022), and *Reticulitermes* spp. (Hongoh et al. 2003), [JQ617833 - Chen et al. unpublished]. However, in all such cases, analogous considerations as above may apply on the potential presence of unrecorded protist hosts, in particular for *A. japonicus* and *Reticulitermes*, in which the gut contents were investigated (Hongoh et al. 2003; Zhao et al. 2022).

To sum up, the vast majority of the representatives of “*Ca*. Intestinibacterium” genus were retrieved from freshwater environments (Figs. 1 and 2A), and the only two well-characterised cases are hosted by protists (Dirren and Posch 2016; present study). Assuming an ancestral association with eukaryotes (which seems reasonable as all characterised present-day *Holosporales* are host-associated bacteria), such observations suggest that freshwater protists could have been the most likely ancestral hosts for the genus “*Ca*. Intestinibacterium”. Under the same line of thought, we may record that the members of the large clade of *Holosporales* encompassing the whole “*Ca*. Paracaedibacteraceae”, “*Caedimonadaceae*”, and *Holosporaceae* families (Fig. 1) were as well typically retrieved in association with freshwater protists, such as amoebozoans, ciliates, dinoflagellates, cercozoans, and euglenids (Horn et al. 1999; Birtles et al. 2000; Baker et al. 2003; Kim et al. 2010; Schulz et al. 2014; Szokoli et al. 2016a; Hess et al. 2016; Schrallhammer et al. 2018; Potekhin et al. 2018; Boscaro et al. 2019; Takeshita et al. 2019; Schrallhammer and Potekhin 2020), with only few exceptions, such as marine diplonemids (Tashyreva et al. 2018), brackish water ciliates (Fokin et al. 2019), and marine (Nunan et al. 2013) and terrestrial arthropods (Konecka and Olszanowski 2019). Thus, these data allow us to speculate on whether even more ancient ancestors of *Holosporales* could have been preferentially associated with freshwater protists. Such a hypothesised reconstruction would possibly distinguish *Holosporales* from the other major alphaproteobacterial lineage of obligate host-associated representatives, namely the *Rickettsiales*, for which the inferred ancestral environment is clearly aquatic (Vannini et al. 2005; Weinert et al. 2009; Wang and Luo 2021), but not yet clarified whether freshwater or marine (Castelli et al. 2022b). Clearly, further investigations, balanced for host phylogeny and ecology, will be necessary to clarify this point.

In conclusion, in this study, we provide the characterisation of “*Ca*. Intestinibacterium parameciiphilum”, a novel species of the “*Ca*. Paracaedibacteraceae” family, living in an intracellular cytoplasmic association with the ciliate *P. biaurelia*. Thus, the known host range and diversity of the so-far strictly endosymbiotic representatives of this family have expanded. In addition, the herein presented results on the environmental distribution of bacteria related to the novel symbiont further demonstrate the added value of screening large 16S rRNA gene metabarcoding datasets (Lanzoni et al. 2019), as well as potentially metagenomic ones (e.g. Schön et al. 2022), as additional sources to investigate the ecology of lineages including typical host-associated bacteria. We underline that such results can be evaluated for inferring still undetermined hosts, and potentially also reconstruct stages of more complex life cycles (e.g. involving multiple hosts). Moreover, considering that the life cycles of these bacteria are understudied and often completely unknown, their consistency with putative host-associated lifestyles can be assessed in comparison with the expectations for free-living conditions, which may still be the most likely prediction for some novel members of otherwise purely host-associated lineages (Schön et al. 2022). In the future, additional multidisciplinary studies, including experimental tests (e.g. Schulz et al. 2014; Potekhin et al. 2018) and genomic analyses (e.g. Garushyants et al. 2018; Castelli et al. 2022a), will help to elucidate the still largely undisclosed diversity, life cycle, and evolutionary history of “*Ca*. Paracaedibacteraceae” and of *Holosporales* in general.

### Emended description of “*Candidatus* Intestinibacterium” Dirren and Posch 2016

“*Candidatus* Intestinibacterium” (*Intestinui*; L. masc. n., internal; bacterium, N.L. neut. n., a rod; N.L. masc. n.; *Intestinibacterium* means rod-shaped bacterium living internal [inside eukaryotic cells]). Gram-negative bacterium, rod-shaped, variable in size. Belongs to the family “*Candidatus *Paracaedibacteraceae” (*Holosporales*, *Alphaproteobacteria*). The type species is “*Ca*. Intestinibacterium nucleariae” (accession number: LN875069; Dirren and Posch 2016). Another species has been described in the cytoplasm of *Paramecium biaurelia*, namely “*Ca*. Intestinibacterium parameciiphilum” (present work). Basis of assignment: positive matching of the FISH 16S rRNA targeting genus-specific probe IntGen_189 (5′-GCGGTAAACCTTTAACCTC-3′).

### Description of “*Candidatus* Intestinibacterium parameciiphilum” sp. nov.

“*Candidatus* Intestinibacterium parameciiphilum” (pa.ra.me.ci.i′phi.lum; N.L. gen. neut. n. paramecii, of the ciliate genus *Paramecium*; N.L. adj. philus -a -um, friend; N.L. neut. adj. *parameciiphilum*, in reference to its natural host *Paramecium*).. Cytoplasmic endosymbiont of the ciliate *Paramecium biaurelia* strain US_Bl 12I1 (Ciliophora, Oligohymenophorea). Short rod-like bacterium (1.50–2.00 × 0.35–0.38 μm in size). Basis of assignment: SSU rRNA gene sequence (accession number: KX702973) and positive match with the specific FISH oligonucleotide probe IntPar_79 (5′-CTAACATATAGAGCAAGCTCC-3′). Belongs to the genus “*Ca*. Intestinibacterium” and the family “*Ca*. Paracaedibacteraceae” (*Holosporales*). Identified in *Paramecium biaurelia* strain US_Bl 12I1 (type strain) isolated from the Skater’s pond, Indiana (USA). Uncultured thus far.

### Supplementary information

ESM 1 Supplementary Table 1 16S rRNA gene sequence similarities for all members of the genus “*Ca.* Intestinibacterium”. The herein characterised “*Ca*. Intestinibacterium parameciiphilum”, as well as identity values within clades, are highlighted in bold. (XLSX 8 kb)
